# Comparison of Ganglion Cell Layer and Ganglion Cell/Inner Plexiform Layer Measures for Detection of Early Glaucoma

**DOI:** 10.1016/j.ogla.2022.06.008

**Published:** 2022-06-30

**Authors:** Golnoush Mahmoudinezhad, Vahid Mohammadzadeh, Jack Martinyan, Kiumars Edalati, Ben Zhou, Dariush Yalzadeh, Navid Amini, Joseph Caprioli, Kouros Nouri-Mahdavi

**Affiliations:** 1 Stein Eye Institute, University of California Los Angeles, Los Angeles, California.; 2 Department of Computer Science, California State University Los Angeles, Los Angeles, California.

**Keywords:** Deviation map, Early glaucoma, Ganglion cell/inner plexiform layer, Ganglion cell layer, Macula

## Abstract

**Purpose:**

To test the hypothesis that macular ganglion cell layer (GCL) measurements detect early glaucoma with higher accuracy than ganglion cell/inner plexiform layer (GCIPL) thickness measurements.

**Design:**

Cross-sectional study.

**Participants:**

The first cohort included 58 glaucomatous eyes with visual field mean deviation (MD) ≥ −6 dB and 125 normal eyes. The second cohort included 72 glaucomatous and 73 normal/glaucoma suspect (GS) eyes with scans able to create GCL/GCIPL deviation maps.

**Methods:**

In the first cohort, 8 × 8 GCL and GCIPL grids were exported and 5 superior and inferior sectors were defined. Global and sectoral GCL and GCIPL measures were used to predict glaucoma. In the second cohort, proportions of scan areas with abnormal (< 5% and < 1% cutoffs) and supernormal (> 95% and > 99% cutoffs) thicknesses on deviation maps were calculated. The extents of GCL and GCIPL abnormal areas were used to predict glaucoma.

**Main Outcome Measures:**

Extents of abnormal GCL/GCIPL regions and areas under receiver operating characteristic curves (AUROC) for prediction of glaucoma were compared between GCL or GCIPL measures.

**Results:**

The average ± standard deviation MDs were −3.7 ± 1.6 dB and −2.7 ± 1.8 dB in glaucomatous eyes in the first and second cohorts, respectively. Global GCIPL thickness measures (central 18° × 18° macular region) performed better than GCL for early detection of glaucoma (AUROC, 0.928 vs. 0.884, respectively; *P* = 0.004). Superior and inferior sector 3 thickness measures provided the best discrimination with both GCL and GCIPL (inferior GCL AUROC, 0.860 vs. GCIPL AUROC, 0.916 [*P* = 0.001]; superior GCL AUROC, 0.916 vs. GCIPL AUROC, 0.900 [*P* = 0.24]). The extents of abnormal GCL regions at a 1% cutoff in the central elliptical area were 17.5 ± 22.2% and 6.4 ± 10.8% in glaucomatous and normal/GS eyes, respectively, versus 17.0 ± 22.2% and 5.7 ± 10.5%, respectively, for GCIPL (*P* = 0.06 for GCL and 0.002 for GCIPL). The extents of GCL and GCIPL supernormal regions were mostly similar in glaucomatous and normal eyes. The best performance for prediction of glaucoma in the second cohort was detected at a *P* value of < 1% within the entire scan for both GCL and GCIPL (AUC, 0.681 vs. 0.668, respectively; *P* = 0.29).

**Conclusions:**

Macular GCL and GCIPL thicknesses are equivalent for identifying early glaucoma with current OCT technology. This is likely explained by limitations of inner macular layer segmentation and concurrent changes within the inner plexiform layer in early glaucoma.

Glaucoma is characterized by progressive loss of retinal ganglion cells (RGCs), whose axons project information to the visual cortex.^1^ The irreversible nature of damage in glaucoma makes early detection of glaucoma crucial.^2^ Glaucoma diagnostic technology has improved significantly in recent years, especially with the availability of automated, computerized analyses of the optic nerve head (ONH) and retinal layers.^3–5^ Spectral-domain OCT (SD-OCT) enables clinicians to closely monitor the health of RGCs and their neural processes—that is, axons and dendrites—by measuring the thickness of the peripapillary retinal nerve fiber layer (RNFL), the neuroretinal rim at the ONH, or inner retinal layers in the macula.^6–8^

It has been hypothesized that macular RGC structural variations could be less frequent in the normal population than variations in RNFL measurements. As a result, early changes in macular RGCs could be distinguished earlier from normal variation in some patients.^9^ Ganglion cell/inner plexiform layer (GCIPL) thickness measurements are able to identify early perimetric glaucoma with a performance approaching that of the RNFL or ONH measurements.^10–12^ Several studies have demonstrated the utility of various macular outcome measures, such as the ganglion cell complex (the combined thicknesses of the macular RNFL, ganglion cell layer [GCL], and inner plexiform layer [IPL]) or the GCIPL (the sum of the GCL and IPL thicknesses), in the detection of glaucoma.^13–15^ However, there are scarce data regarding the role of the GCL for this purpose.^16–18^

The updated version of Spectralis OCT’s Glaucoma Module Premium Edition (GMPE) uses the proprietary Anatomic Positioning System (APS) and provides deviation maps for both the GCL and GCIPL compared with normative data for the central 30° × 25° macular region. Hence, the performances of these 2 layers can now be compared with regard to detection of abnormal macular regions in glaucomatous and glaucoma suspect (GS) eyes. The aim of this study is to compare the performance of the macular GCL and GCIPL layers for detection of early signs of glaucoma using the following: (1) the conventional macular thickness measures; and (2) the newly available macular GCL and GCIPL deviation maps on Spectralis OCT in 2 cohorts of patients with glaucoma and subjects with normal eyes.

## Methods

This cross-sectional observational study included 2 cohorts. The first cohort consisted of 58 eyes (58 patients) with established glaucoma and 125 normal eyes (73 patients). The second cohort included 28 eyes from 25 normal subjects, 45 eyes from 41 GS subjects, and 72 glaucomatous eyes from 63 patients. The study was approved by the Human Research Protection Program at the University of California Los Angeles, which waived the requirement for informed consent for this study. All study procedures adhered to the tenets of the Declaration of Helsinki and the Health Insurance Portability and Accountability Act.

The inclusion criteria for glaucomatous eyes in this study were as follows: (1) a clinical diagnosis of primary open-angle glaucoma, primary angle-closure glaucoma, pseudoexfoliative glaucoma, or pigmentary glaucoma; (2) the availability of good-quality Spectralis macular volume scans (quality factor > 15); or (3) the absence of confounding macular pathologies, such as an epiretinal membrane, diabetic retinopathy, or age-related macular degeneration, based on a subjective review of the images. Segmentation of macular layers was checked and corrected manually if required.

Cohort 1 glaucomatous eyes had both glaucomatous discs, as diagnosed by an attending physician (K.N.M.), and abnormal visual fields (VFs), confirmed once. Cohort 2 glaucomatous eyes were required to have either glaucomatous discs or abnormal VFs (see above). Optic disc photographs from this cohort were reviewed by 2 glaucoma experts (K.N.M. and J.C.) individually; in case of a disagreement, the 2 glaucoma specialists reviewed the optic disc photographs together and reached a consensus. Eyes with a glaucomatous-appearing optic disc and/or glaucomatous VF loss were considered to have glaucoma.^19^ The normal control eyes had intraocular pressure (IOP) ≤ 21 mmHg with no history of elevated IOP, normal-appearing optic discs with intact neuroretinal rims, and normal VF test results. The GS group included eyes with suspicious-appearing optic nerves based on the review of stereoscopic ONH photographs, with or without high IOP (> 21 mmHg), and no evidence of repeatable glaucomatous VF damage.^20^ Both OCT and VF tests were required to be available within 6 months from each other in both cohorts.

### OCT Imaging and Perimetry

**Figure 1.**
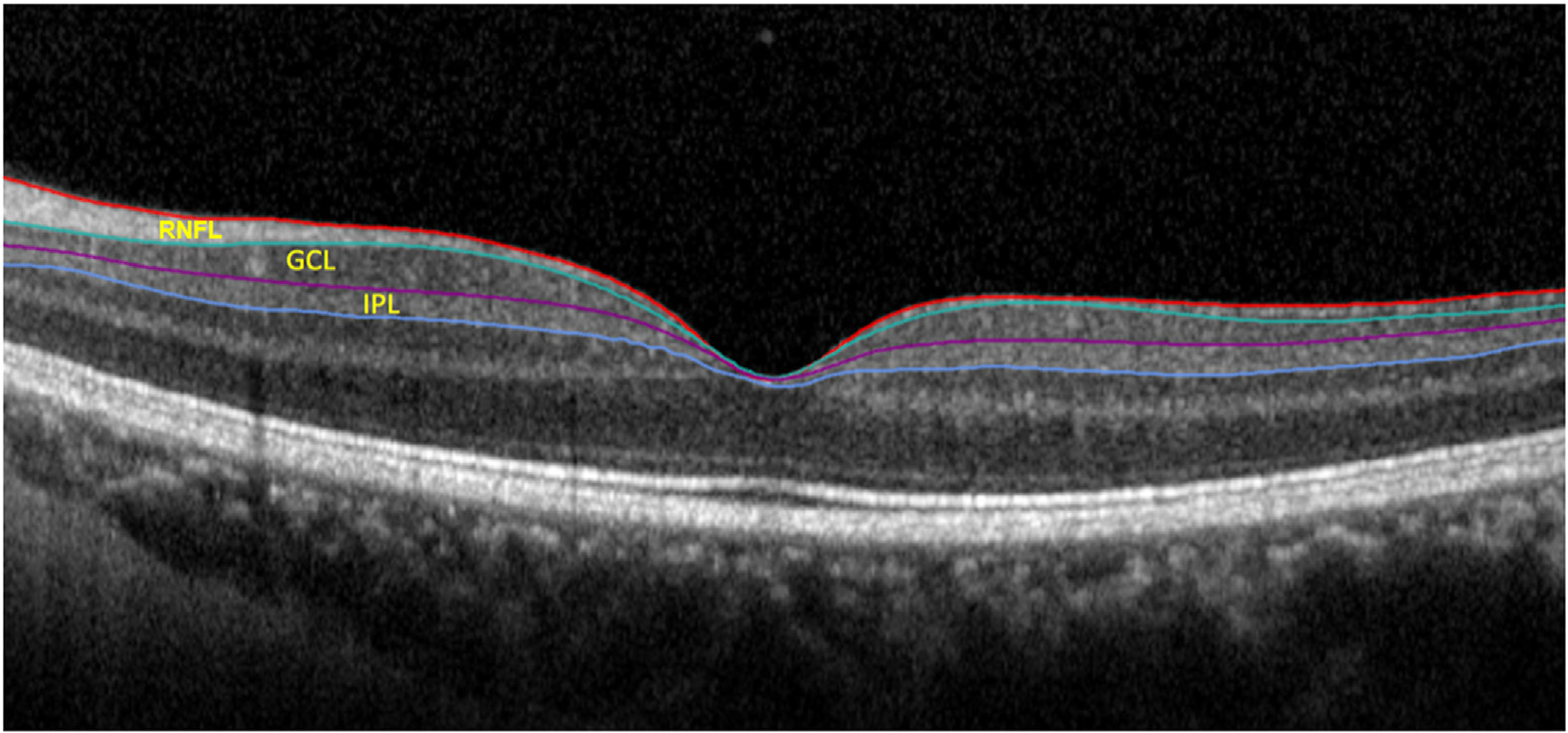
An example of segmentation of inner retinal layers of interest in glaucoma by the Glaucoma Module Premium Edition software. GCL = ganglion cell layer; IPL = inner plexiform layer; RNFL = retinal nerve fiber layer.

**Figure 2.**
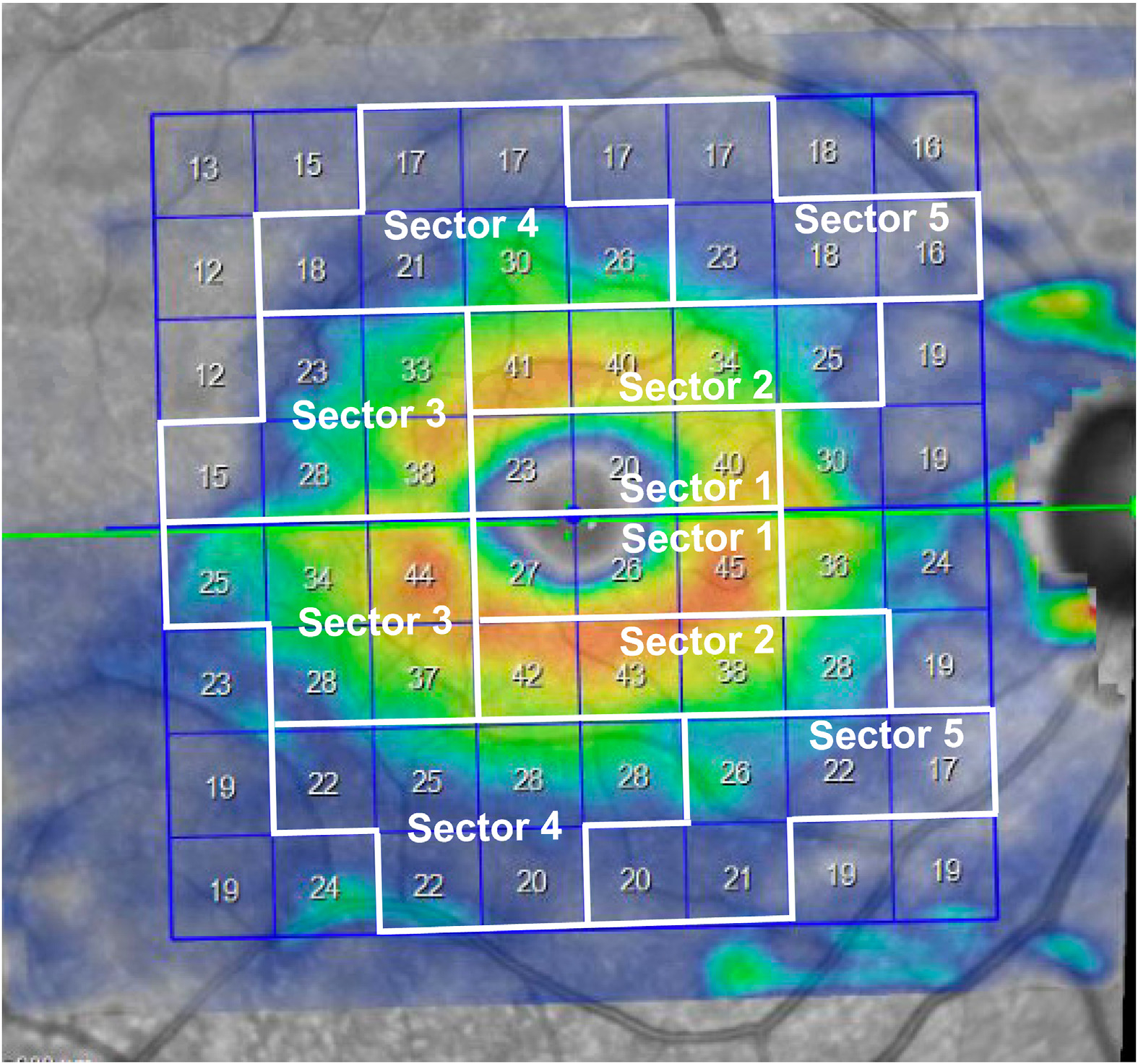
An example of a color map for ganglion cell layer thickness as an 8 × 8 grid based on the Posterior Pole Algorithm of the Spectralis spectraldomain OCT. The central 48 superpixels are grouped in 5 superior and inferior sectors according to Um et al.^21^

All recruited eyes underwent macular imaging with Spectralis SD-OCT. The Spectralis OCT volume scan is composed of 61 B-scans (each including 768 A-scans), which are acquired along the fovea-disc centroid axis. Each scan is repeated 9 to 11 times to decrease the speckle noise and provide the best quality. The GMPE software was used to segment retinal layers and measure GCL and IPL thicknesses (Figure 1). The data were exported in an 8 × 8 grid of 64 superpixels, which are centered on the fovea. In the first cohort, we defined 5 superior and 5 inferior macular sectors and averaged GCL and GCIPL thickness measurements in each of the 5 sectors (Fig 2).^21^

**Figure 3.**
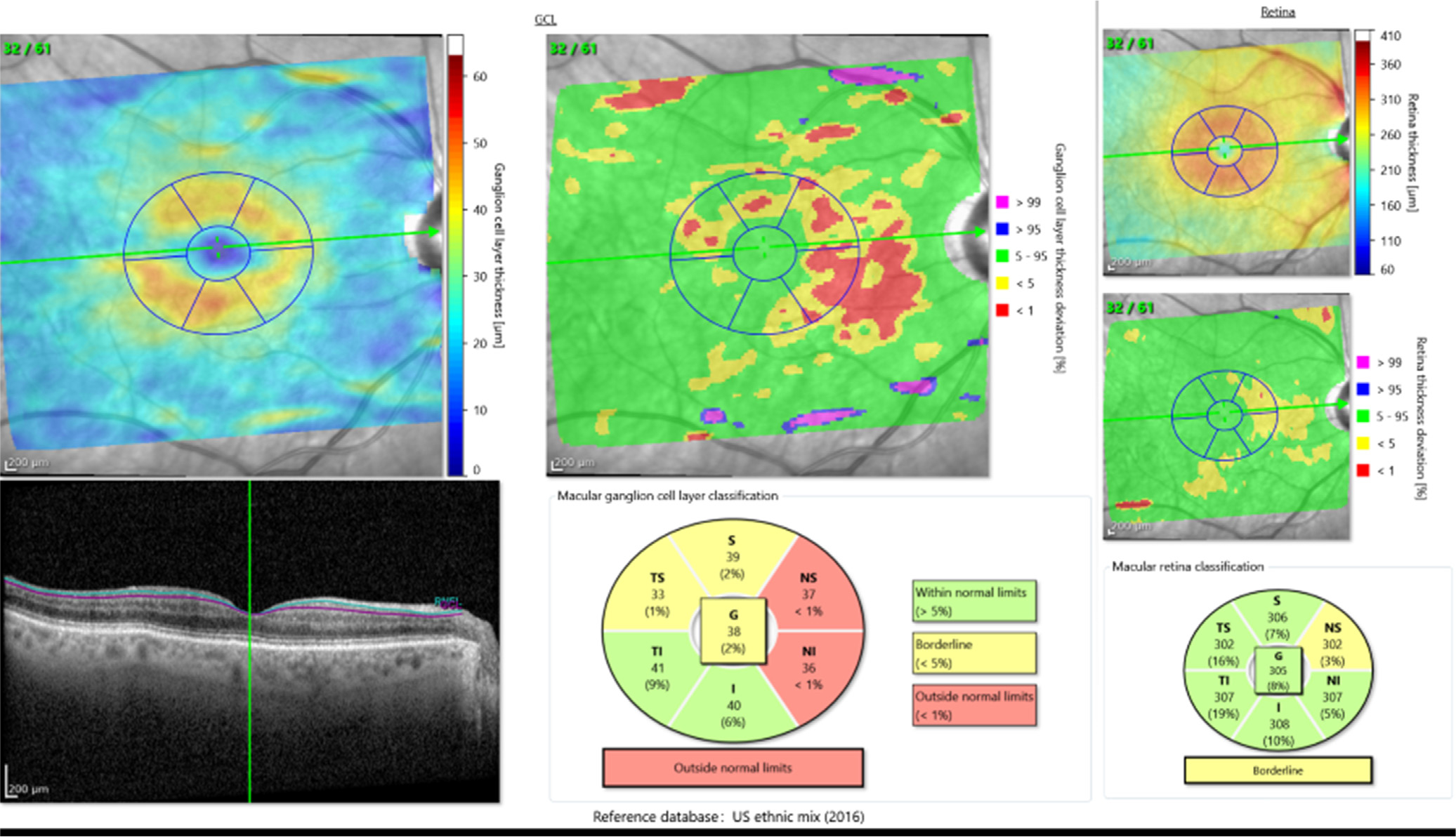
The Glaucoma Module Premium Edition uses the proprietary Anatomic Positioning System for acquiring macular scans along the fovea-Bruch membrane axis and provides ganglion cell layer and ganglion cell/inner plexiform layer deviation maps. On the deviation map, abnormal areas are flagged as red (*P* < 1%) and yellow (*P* < 5%). Supernormal areas are annotated as pink (*P* > 99%) and blue (*P* > 97%). GCL = ganglion cell layer; RNFL = retinal nerve fiber layer; T = temporal; TS = temporal superior; TI = temporal inferior; G = global; I = inferior; NS = nasal superior; NI = nasal inferior.

The most recent GMPE software uses macular scans acquired with the proprietary APS to align the volume scan along the fovea-Bruch membrane axis. A research software program provided by Heidelberg Engineering was used to create GCL and GCIPL deviation maps (Fig 3). Each pixel is compared with the normative database and marked as within normal limits (green) if it falls within the 5% to 95% prediction interval of the normative database. Pixels with decreased thicknesses are flagged as borderline abnormal (*P* < 5%; yellow) or abnormal (*P* < 1%; red). Supernormal pixels are marked as blue (*P* > 95%) or pink (*P* > 99%; Fig 3). We quantified abnormal and supernormal regions on the deviation maps by counting the number of pixels for each color and dividing it by the total number of pixels in the entire rectangular scan area. Similarly, such abnormal and supernormal regions were quantified in an elliptical annulus around the fovea that was 4.8 × 4.0 mm in size, excluding a central oval area 1.2 × 1.0 mm in diameter that corresponds to the foveola. According to histologic and OCT studies of the human retina, the highest density of ganglion cells resides in this area.^22,23^ To detect the elliptical annulus, the deviation map image was converted to a grayscale image on which a least-squares criterion was applied to estimate the best fit to the outer and inner elliptical boundaries. Furthermore, a Hough transform was applied to eliminate the pixels that reside on the straight lines that define the sectors within the elliptical annulus.

Only VFs with false-positive rates < 15% were included. Perimetry was carried out with the Humphrey Field Analyzer’s standard Swedish Interactive Thresholding Algorithm 24–2 (Carl Zeiss Meditec). An abnormal VF was defined as: (1) a pattern standard deviation (SD) with a *P* value of < 0.05; (2) a Glaucoma Hemifield Test result that was outside normal limits; or (3) 3 or more abnormal points with a probability of a *P* value of < 0.05, of which at least 1 point had a *P* value of < 0.01 in the pattern deviation map.^19^ Visual fields not meeting the above criteria were considered to be normal.

### Statistical Analyses

Outcomes of interest for the first cohort were comparisons of global and regional average GCL and GCIPL thicknesses in each of the 5 sectors in groups with glaucomatous and normal eyes and discrimination of glaucoma from normal eyes (Fig 2). Areas under receiver operating characteristic curves (AUROCs) were used to compare the performance of various outcome measures of interest. In the second cohort, to compare the performance of GCL versus GCIPL using a deviation map, the proportion of areas with abnormal (5% and 1% cutoffs) and supernormal (95% and 99% cutoffs) thicknesses were estimated on the macular deviation maps with a proprietary algorithm written in matrix laboratory (MATLAB) (Fig 3). We compared the extents of abnormal (to check for sensitivity) and supernormal regions in the normal/GS versus glaucomatous eyes with Wilcoxon test and used AUROCs to compare the performance of the GCL and GCIPL abnormal areas for the prediction of early glaucoma. We compared receiver operating characteristic curves with a nonparametric test using the method described by DeLong et al.^24^

## Results

The average ± SD 24–2 VF mean deviations (MDs) in the glaucomatous group were −3.7 ± 1.6 dB in the first cohort and −2.7 ± 1.8 dB in the second cohort. The average ± SD MDs for normal eyes in the first and second cohorts were −0.8 ± 1.5 dB and −0.9 ± 1.5 dB, respectively. Table 1 describes the demographic and clinical characteristics of the 2 cohorts.

**Figure 4.**
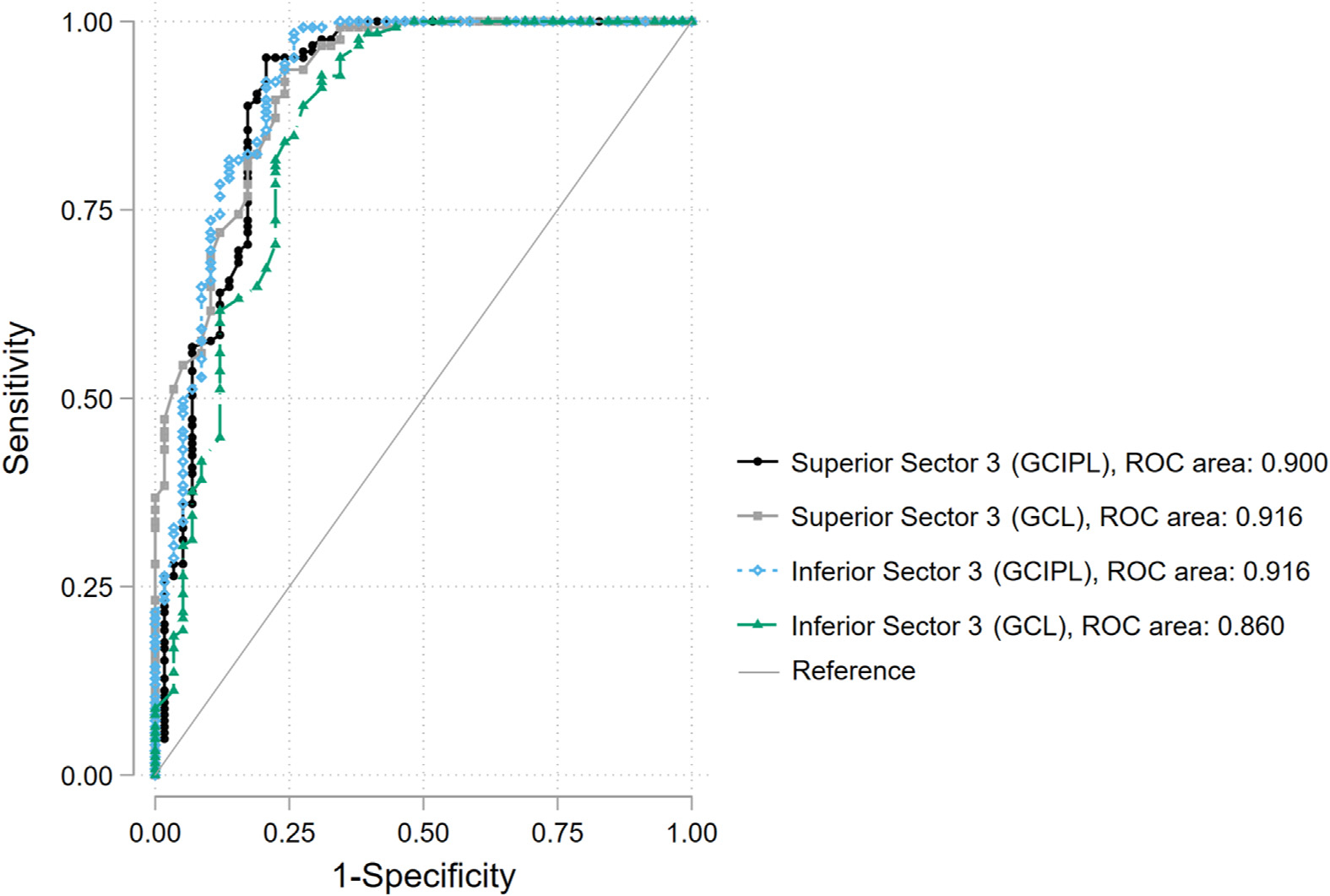
Comparison of the receiver operating characteristic curves for the best-performing measures for ganglion cell/inner plexiform layer and ganglion cell layer. GCIPL = ganglion cell/inner plexiform layer; GCL = ganglion cell layer; ROC = receiver operating characteristic.

In the first cohort, the average macular GCIPL thickness (50.6 ± 5.7 μm) and GCL thickness (24.3 ± 2.5 μm) in the central 18° × 18° region were thinner in the glaucomatous eyes as compared with the normal eyes (62.2 ± 6.2 μm and 28.4 ± 2.9 μm; *P* < 0.001). Table 2 summarizes the AUROCs for global and sectoral GCL and GCIPL thicknesses for discrimination of glaucoma from normal eyes in the first cohort. The highest AUROCs for prediction of glaucoma were observed in inferior sector 3 for GCIPL (0.916) and 0.860 for GCL (*P* = 0.001) and superior sector 3 (GCIPL AUROC, 0.916; GCL AUROC, 0.900; *P* = 0.24). The AUROCs for the global macular thickness (central 18° × 18° area) for discriminating between normal and glaucomatous eyes were 0.928 and 0.884 for GCL and GCIPL, respectively (*P* = 0.004). Figure 4 compares the receiver operating characteristic curves for the inferior and superior sectors 2 and 3.

Table 3 demonstrates a comparison of the extent of the abnormal (*P* < 1% and *P* < 5%) and supernormal (*P* > 95% and *P* > 99%) areas on the macular deviation maps for GCL and GCIPL thicknesses in the normal/GS and glaucomatous groups in both the entire scan and central elliptical regions. In the central elliptical area, the average ± SD extents of GCL damage in glaucomatous and normal/GS eyes at 1% cutoffs were 17.5 ± 22.2% and 6.4 ± 10.8%, respectively, versus 17.0 ± 22.2% and 5.7 ± 10.5% for GCIPL, respectively (*P* = 0.06 and 0.002). The extent of GCL damage at a *P* value of < 5% was not different from that of GCIPL in the glaucoma (*P* = 0.15) and normal/GS eyes (*P* = 0.88), respectively. The extents of GCL and GCIPL supernormal regions were similar in the 2 groups at > 95% (*P* = 0.81 and 0.13) and > 99% cutoffs, respectively (*P* = 0.98 and 0.12, respectively).

**Figure 5.**
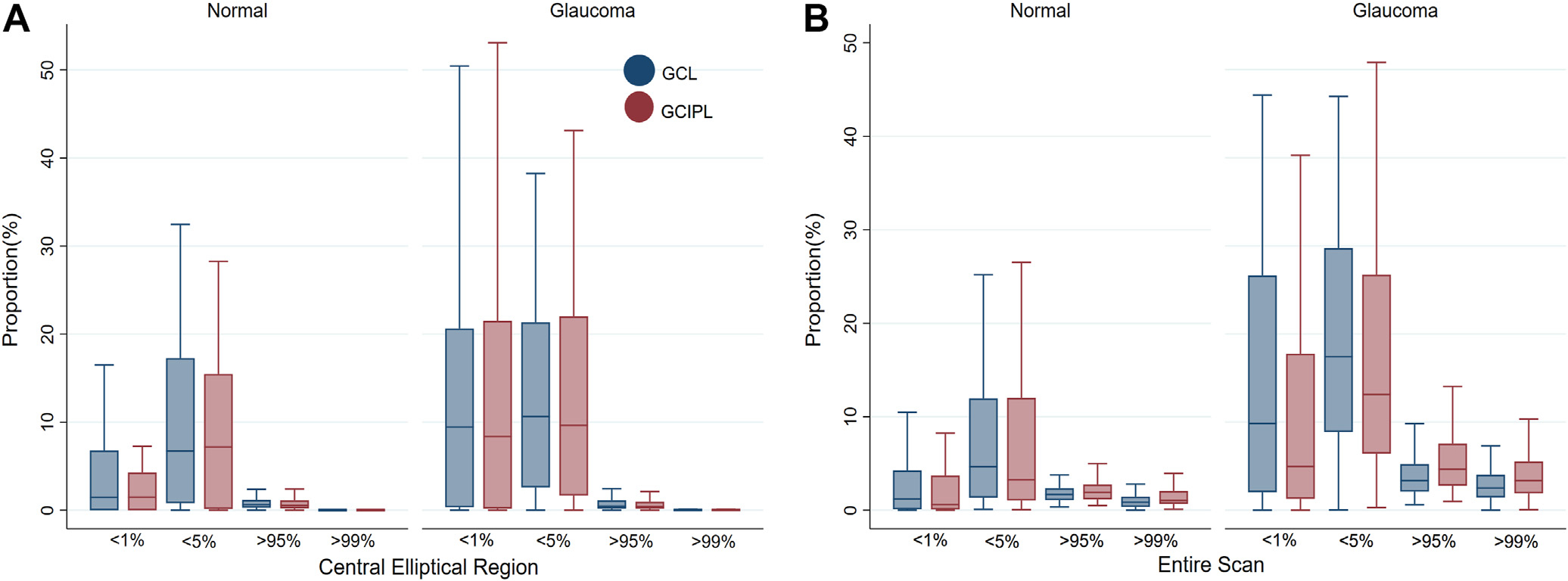
Comparison of the extents of the abnormal ganglion cell layer and ganglion cell/inner plexiform layer regions (*P* values < 5% and < 1%) and supernormal areas (*P* values > 95% and > 99%) on the macular deviation maps in normal and glaucoma eyes (cohort 2) within (**A**) the central elliptical region and (**B**) the entire scan area. GCIPL = ganglion cell/inner plexiform layer; GCL = ganglion cell layer.

The extents of GCL abnormalities, as compared with GCIPL abnormalities, at 5% (*P* = 0.001) and 1% (*P* < 0.001) cutoffs and at 95% (*P* < 0.001) and 99% (*P* < 0.001) cutoffs were significantly different in the entire scan in glaucomatous eyes, with GCL detecting a slightly higher extent of abnormalities and a slightly lower extent of supernormal regions. In normal eyes, the extent of GCL abnormalities was higher as compared with the extent of GCIPL abnormalities, both at 5% (*P* = 0.04) and 1% (*P* < 0.001) cutoffs, while, at the same time, the extents of the supernormal region were lower at 95% (*P* < 0.001) and 99% (*P* < 0.001) cutoffs: that is, GCL detected a higher extent of abnormalities and a lower area of supernormal pixels in normal eyes. A comparison of the extents of abnormal and supernormal regions in glaucomatous and normal eyes in the entire scan and in the central elliptical region is presented in Figure 5. There were no significant differences between GCL and GCIPL with regard to discrimination of early glaucoma from normal/GS eyes in the second cohort, and the AUROCs were uniformly low (Table 4). The highest performance for detection of early glaucoma was at *P* values of < 1% in the entire scan for both GCL and GCIPL (AUROC, 0.681 vs. 0.668, respectively; *P* = 0.29).

## Discussion

Diagnosis of glaucoma in early stages and recognition of disease progression remain challenging tasks in need of optimization. Macular OCT imaging has become a standard approach to assess the central RGCs. Segmentation of individual retinal layers is now possible with the current level of resolution achieved with OCT imaging in the clinical setting. However, the boundary between GCL and IPL is less well demarcated on OCT imaging than are boundaries between the other macular layers. For this reason, some OCT devices provide the GCIPL or ganglion cell complex (GCC) thicknesses as the main macular outcome measures for glaucoma diagnostics. Most prior OCT studies focused on assessing the GCIPL or GCC thickness rather than the GCL or IPL thickness individually.^25,26^ Ganglion cell/inner plexiform layer and GCC thickness measurements display strong structure-function correlations with perimetric measures in early glaucoma.^23,27,28^ Spectralis OCT provides GCL thickness as the macular layer of choice for glaucoma diagnostics based on the assumption that changes in the GCL proper would be most sensitive for the detection of early damage in glaucomatous eyes. Few studies have evaluated GCL thickness measurements for the detection of early glaucoma.^3,14,29,30^

We compared the performance of GCIPL and GCL using both thickness measurements (cohort 1) and deviation maps (cohort 2) to test the hypothesis that the GCL thickness, which mostly includes the RGC somas, is superior to GCIPL for discrimination of patients with glaucoma from those with normal or GS eyes. The GCL thickness showed comparable accuracy to the GCIPL thickness for the discrimination of early glaucomatous eyes from normal eyes in the first cohort based on global or sectoral macular thickness measures. In the second cohort, statistically larger areas of GCL abnormalities were detected in the glaucoma group compared with areas of GCIPL abnormalities. However, this finding was also true in the normal/GS group. Most importantly, the differences were not large enough to be considered clinically relevant in either the central elliptical region or the entire macular scan. Our findings demonstrate that despite minor differences, GCIPL and GCL measures perform similarly for identifying early, structural glaucoma damage, and there is no significant benefit to measuring the GCL thickness in isolation with the current level of OCT technology. Given the criteria used by the OCT device, approximately 5% of pixels would be expected to be flagged at the 5% and 95% cutoff points. We measured the supernormal regions of the macula in order to have an understanding of the performance of the deviation maps at both ends of the abnormality spectrum.

Two prior studies evaluated the performance of various macular retinal layers for glaucoma diagnoses, including GCL and GCIPL, and reported comparable diagnostic accuracy between GCL and GCIPL.^14,30^ However, Pazos et al^3^ found higher accuracy for GCIPL as compared with GCL for the discrimination of glaucomatous from healthy eyes. In contrast, Chua et al^29^ reported higher accuracy (AUROC, 0.842) in detecting eyes with early glaucoma with macular GCL compared with GCIPL. Springelkamp et al^31^ demonstrated that the mean GCL thickness in the inferior half of the macular region showed the highest AUROC (0.85; 95% confidence interval, 0.77–0.92) and sensitivity (54%; 95% confidence interval, 39%–68%) using the Topcon OCT device and was the best-performing measure among a number of macular thickness measures. Nouri-Mahdavi et al^11^ found that the average inferior GCIPL had the highest diagnostic accuracy for detection of early perimetric glaucoma (AUROC, −0.94). We used sectors introduced by Um et al^21^ for defining 5 superior and inferior macular sectors in our first cohort. Although the sectors introduced by Um et al^21^ have not been validated, the concept of such macular sectors follows that of the Glaucoma Hemifield Test, as central VF points and OCT superpixels tend to follow the patterns of macular RNFL bundles.^32,33^ The performance of the superior and inferior sectoral GCL and GCIPL thickness measures was similar to the above studies for the detection of early perimetric glaucoma, and the differences in performance between GCL and GCIPL were not clinically relevant among different sectors. In this study, the best GCIPL thickness measure, average thickness in the central 18° × 18° macular region, had an AUC of 0.928 with a corresponding AUROC of 0.884 for the same GCL region (*P* = 0.004). Interestingly, the best-performing GCL measure was the superior sector 3, with an AUROC of 0.916, compared with 0.900 for GCIPL (*P* = 0.24).

We used a second cohort of glaucomatous and normal/GS eyes that had undergone imaging with the APS of the Spectralis OCT to be able to assess and compare the performance of macular deviation maps. Only images acquired with this newer software are able to be analyzed to create deviation maps. We found a slightly higher proportion of abnormal superpixels across the macula for the GCL than the GCIPL and a slightly lower proportion of supernormal superpixels, regardless of the *P* value cutoff, in the entire scan in glaucomatous eyes. Although some of these comparisons were statistically significant because of the very large number of superpixels in each image, the clinical relevance of these findings is questionable. In addition, GCL still identified a slightly higher proportion of abnormal superpixels compared with GCIPL in the normal/GS group, which we included as a test of specificity, either in the entire scan or the central elliptical region. Conversely, the GCIPL maps tended to show somewhat higher rates of supernormal superpixels in the normal/GS group. Given the very small differences for both sets of comparisons, we do not believe that the observed differences have any clinical relevance.

The diagnostic accuracy of macular measures such as GCL also depends on the location of the region of interest; for example, Kim et al^18^ reported an AUROC of 0.695 for the GCL thickness in the 1 to 2 mm macular region compared to an AUROC of 0.852 in the 3 to 6 mm region. Larger macular scans perform better with regards to the detection of glaucoma, likely because of the fact that inclusion of a larger peripheral macular region adds important additional information.^25,34^ In comparison, averaging thickness measures in larger macular sectors could decrease the variability observed in smaller sectors and superpixels and, hence, could improve performance, depending on the study cohort.^14,26,35,36^ In our study, the global 18° × 18° macular GCIPL thickness was the best measure for the detection of early perimetric glaucoma (AUC, 0.928), with comparable but lower GCL performance (AUC, 0.884).

The AUROCs for discrimination of glaucoma based on the areas of pixel abnormalities were much lower for both GCL and GCIPL thickness measures in cohort 2 compared with cohort 1, demonstrating poor performance for discrimination of early glaucomatous eyes from a group of normal or GS eyes (Table 4). The best-performing measure for both the GCL and GCIPL was the extent of abnormality at a *P* value of < 1% in the entire scan (AUC, 0.681 vs. 0.668, respectively; *P* = 0.29). Two features of the study design at least partially explain this discrepancy in performance. Glaucoma was defined based on either a VF or disc abnormality in cohort 2 rather than on both VFs and discs, as in cohort 1. This led to the inclusion of milder glaucoma cases compared with cohort 1, as reflected in the higher (better) MD for cohort 2 (MD = −3.7 ± 1.6 dB in cohort 1 vs. −2.7 ± 1.8 dB in cohort 2). In addition, the GS and normal eyes were grouped together to create a larger control group for cohort 2. This could have led to the misclassification of some eyes with very early glaucoma in the control group and resulted in lower classification accuracy. Many prior studies categorized eyes with only disc abnormalities and normal VFs as GSs. Formal testing of various OCT thickness measures with regard to the detection of glaucoma in such eyes showed lower performance for most such OCT measures, including macular thickness measures.^12,37^ The lower performance of GCL as compared with GCIPL could also be explained by segmentation and alignment errors in the inner macular layer,^38,39^ as well as concurrent changes within the IPL in early glaucoma.

The limitations of the current study need to be taken into account. Although OCT measures demonstrate good diagnostic abilities for single parameters in early glaucoma, they may either falsely classify healthy eyes as having glaucoma or miss the diagnosis of early glaucoma in a small proportion of subjects.^40,41^ A recent report by Tsamis et al^42^ showed that a detection method that relies solely on the use of single OCT or VF metrics may lead to the misidentification of some healthy control eyes as glaucomatous or fail to detect some eyes with early glaucomatous damage. The number of eyes was smaller in cohort 2, leading to low power to detect significant differences, if they existed. However, the differences between the AUROCs for GCL and GCIPL measures in the second cohort were very small and not relevant clinically. Combining normal and GS eyes in cohort 2 assumes that GS eyes share similar characteristics with healthy eyes. However, this may have led to low discriminatory power between glaucomatous eyes and the control group, because some GS eyes may have had subtle early glaucoma damage. Future studies are needed to look into the discriminatory power of GCL versus GCIPL in healthy eyes with no structural changes versus early glaucomatous eyes. We also manually corrected the segmentation of macular OCT scans, as suggested by the manufacturer. This might have resulted in overestimation of the performance of OCT metrics in this study. In actual clinical practice, correction of segmentation is not routinely done.

In conclusion, we found that GCL and GCIPL thicknesses perform similarly for the detection of early glaucoma damage. Their performance was significantly worse if eyes with preperimetric glaucoma were included. The lower performance of GCL as compared with GCIPL in some of the analyses could also be explained by segmentation and alignment errors in the inner macular layer, as well as concurrent changes within the inner plexiform layer in early glaucoma. Both GCL and GCIPL measures may be used effectively for identifying early macular structural damage with the current SD-OCT technology.

## Figures and Tables

**Table 1.** Demographic and Clinical Characteristics of the First and Second Cohorts

	First Cohort			Second Cohort		
	
Variable	*Normal*	*Glaucoma*	P *Value*	*Normal/Suspect*	*Glaucoma*	P *Value*

Eyes/patients, n	125/73	58/58		73/66	72/63	
Laterality, right (%)/left (%)	69 (55.2)/56 (44.8)	29 (50.0)/29 (50.0)	0.51	32 (43.8)/41 (56.2)	30 (41.7)/42 (58.3)	0.8
Gender, female (%)/male (%)	39 (53.4)/34 (46.6)	38 (65.5)/20 (34.5)	0.09	33 (63.5)/19 (36.5)	29 (50.9)/28 (49.1)	0.01
Age ± SD, yrs	53.1 ± 13.8	65.6 ± 9.4	< 0.001	65.0 ± 13.6	71.4 ± 11.8	0.003
Visual acuity ± SD, logMAR	0.02 ± 0.08	0.07 ± 0.09	< 0.001	0.08 ± 0.1	0.1 ± 0.1	0.24
Intraocular pressure ± SD, mmHg	13.8 ± 2.7	13.3 ± 4.3	0.049	15.6 ± 3.6	13.8 ± 3.5	0.003
Visual field MD ± SD, dB	−0.8 ± 1.5	−3.7 ± 1.6	< 0.001	−0.9 ± 1.5	−2.7 ± 1.8	< 0.001

LogMAR = logarithm of the minimum angle of resolution; MD = mean deviation; SD = standard deviation.

**Table 2.** Areas under the Receiver Operating Characteristic Curves of Macular Layer Thickness Parameters (Ganglion Cell Layer vs. Ganglion Cell/Inner Plexiform Layer) for Detection of Early Glaucoma

		Area Under ROC Curve		
Sectoral Regions		*GCL*	*GCIPL*	*P* Value

Sector 1	Superior	0.798	0.799	0.97
	Inferior	0.762	0.746	0.43
Sector 2	Superior	0.857	0.864	0.57
	Inferior	0.820	0.845	0.10
Sector 3	Superior	0.916	0.900	0.24
	Inferior	0.860	0.916	0.001
Sector 4	Superior	0.851	0.898	0.009
	Inferior	0.689	0.787	< 0.001
Sector 5	Superior	0.793	0.752	0.1
	Inferior	0.511	0.582	0.02
Central 18° × 18° (global)	NA	0.884	0.928	0.004

GCIPL = ganglion cell/inner plexiform layer; GCL = ganglion cell layer; ROC = receiver operating characteristic; NA = not applicable.

**Table 3.** Comparison of the Extents of Abnormal and Supernormal Areas on Macular Deviation Maps for Ganglion Cell Layer and Ganglion Cell/Inner Plexiform Layer at Various Cutoff Points in the Entire Macular Scan and the Central Elliptical Regions

	Normal/Suspect Eyes			Glaucoma Eyes		
		
	*GCL*, %	*GCIPL*, %	P *Value*	*GCL*, %	*GCIPL*, %	P *Value*

*P* < 1% (central ellipse)	6.4 ± 10.8	5.7 ± 10.5	0.002	17.5 ± 22.2	17 ± 22.2	0.06
*P* < 5% (central ellipse)	10.4 ± 10.9	10.2 ± 11.2	0.88	12.8 ± 11.2	12.5 ± 11.8	0.15
*P* < 1% (entire scan)	3.4 ± 5.1	2.6 ± 4.0	< 0.001	9.2 ± 11.6	6.6 ± 9.1	< 0.001
*P* < 5% (entire scan)	7.1 ± 6.7	6.6 ± 7.1	0.04	9.49 ± 6.6	8.7 ± 7.3	0.001
*P* > 95% (central ellipse)	2.2 ± 5.2	2.0 ± 4.9	0.13	1.9 ± 4.1	2.0 ± 4.3	0.81
*P* > 99% (central ellipse)	1.6 ± 8.3	1.9 ± 9.7	0.12	0.4 ± 1.9	0.3 ± 1.1	0.98
*P* > 95% (entire scan)	2.5 ± 3.5	2.8 ± 3.9	< 0.001	2.4 ± 2.3	3.0 ± 2.5	< 0.001
*P* > 99% (entire scan)	2.4 ± 6.4	3.0 ± 7.4	< 0.001	2.0 ± 2.9	2.6 ± 3.3	< 0.001

GCIPL = ganglion cell/inner plexiform layer; GCL = ganglion cell layer.

**Table 4.** Areas under Receiver Operating Characteristic Curves for Detection of Early Glaucoma from Normal/Suspect Eyes in Cohort 2*

	GCL	GCIPL	*P* Value

*P* < 1% (central ellipse)	0.658	0.666	0.50
*P* < 5% (central ellipse)	0.572	0.564	0.54
*P* < 1% (entire scan)	0.681	0.668	0.29
*P* < 5% (entire scan)	0.615	0.597	0.14

GCIPL = ganglion cell/inner plexiform layer; GCL = ganglion cell layer.

* The areas under receiver operating characteristic curves were estimated based on the extents of the abnormal areas on macular deviation maps at different cutoff points (*P* < 1% and < 5%).

## Abbreviations and Acronyms:

APS: Anatomic Positioning System
AUROC: area under the receiver operating characteristic curve
GCIPL: ganglion cell/inner plexiform layer
GCL: ganglion cell layer
GMPE: Glaucoma Module Premium Edition
GS: glaucoma suspect
IOP: intraocular pressure
IPL: inner plexiform layer
MD: mean deviation
RGC: retinal ganglion cell
ROC: receiver operating characteristic
RNFL: retinal nerve fiber layer
SD: standard deviation
SD-OCT: spectral-domain OCT
VF: visual field
